# Multiple Stressor Effects of Radon and Phthalates in Children: Background Information and Future Research

**DOI:** 10.3390/ijerph17082898

**Published:** 2020-04-22

**Authors:** W. S. Kwan, D. Nikezic, Vellaisamy A. L. Roy, K. N. Yu

**Affiliations:** 1 Department of Physics, City University of Hong Kong, Tat Chee Ave, Kowloon Tong, Kowloon, Hong Kong, China; wingskwan8@cityu.edu.hk; 2 Department of Materials Science and Engineering, City University of Hong Kong, Tat Chee Ave, Kowloon Tong, Kowloon, Hong Kong, China; 3 Department of Mathematical Sciences, State University of Novi Pazar, Vuka Karadžića 9, RS-36300 Novi Pazar, Serbia; nikezic@kg.ac.rs; 4 Faculty of Science, University of Kragujevac, R. Domanovica 12, 34000 Kragujevac, Serbia; 5 James Watt School of Engineering, University of Glasgow, Glasgow G12 8QQ, UK; 6 State Key Laboratory in Marine Pollution, City University of Hong Kong, Tat Chee Ave, Kowloon Tong, Kowloon, Hong Kong, China

**Keywords:** multiple stressor effects, radon, phthalates, children, dosimetric modeling

## Abstract

The present paper reviews available background information for studying multiple stressor effects of radon (^222^Rn) and phthalates in children and provides insights on future directions. In realistic situations, living organisms are collectively subjected to many environmental stressors, with the resultant effects being referred to as multiple stressor effects. Radon is a naturally occurring radioactive gas that can lead to lung cancers. On the other hand, phthalates are semi-volatile organic compounds widely applied as plasticizers to provide flexibility to plastic in consumer products. Links of phthalates to various health effects have been reported, including allergy and asthma. In the present review, the focus on indoor contaminants was due to their higher concentrations and to the higher indoor occupancy factor, while the focus on the pediatric population was due to their inherent sensitivity and their spending more time close to the floor. Two main future directions in studying multiple stressor effects of radon and phthalates in children were proposed. The first one was on computational modeling and micro-dosimetric studies, and the second one was on biological studies. In particular, dose-response relationship and effect-specific models for combined exposures to radon and phthalates would be necessary. The ideas and methodology behind such proposed research work are also applicable to studies on multiple stressor effects of collective exposures to other significant airborne contaminants, and to population groups other than children.

## 1. Introduction to Multiple Stressor Effect

**Figure 1:**
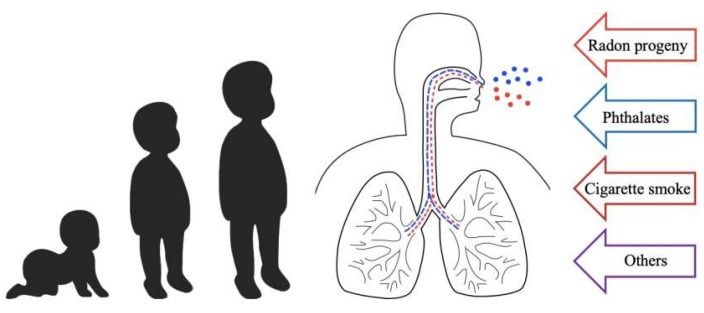
Multiple stressor effects on human beings caused by collective exposure to various significant airborne contaminants, e.g., radon progeny, phthalates, cigarette smoke, etc.

In reality, it is inevitable that living organisms are subjected to various environmental stressors collectively, with the resultant effects being referred to as multiple stressor effects, and the assessment on the probability and seriousness of the resultant effects is referred to as cumulative risk assessment [1]. For realistic cumulative risk assessments, therefore, thorough understanding on the multiple stressor effects is indispensable [2,3,4,5,6,7,8]. As such, when discussing the effects of airborne contaminants on human beings, the effects from collective exposures to all significant airborne contaminants (e.g., ionizing radiation, chemicals, cigarette smoke, heavy metals, etc.) would be relevant, as depicted in Figure 1.

In ecological risk assessment nowadays, the radionuclides emitting ionizing radiations and other types of airborne contaminants are usually separately regulated, which has effectively assumed no interactive effects between ionizing radiation and other airborne contaminants. However, the resultant toxicity could depend on the simultaneous or sequential exposures [9,10]. In relation, Mothersill et al. cautioned that it would be complicated to predict the resultant effects or to determine the safe levels [4], while Sexton and Hattis also pointed out problems that might hamper the cumulative risk assessment [1].

**Figure 2:**
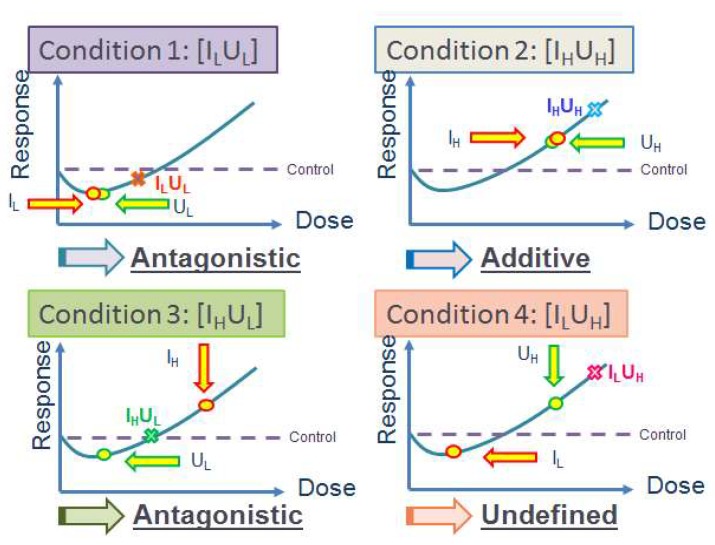
Multiple stressor effects from studies on combined effects of α-particles and depleted uranium (DU) on zebrafish *(Danio rerio)* embryos [15,16]. Conditions 1 to 4: [I_L_] and [I_H_] refer to low and high α-particle doses, respectively; [U_L_] and [U_H_] refer to low and high DU exposures, respectively. Circles: effects from individual stressors; crosses: effects from multiple stressors.

In reality, multiple stressor effects are not necessarily a simple sum of the effects from individual stressors [10,11], but can be represented by additive, synergistic, antagonistic, or even more complicated relationships. Our group previously performed a series of studies on multiple stressor effects on zebrafish *(Danio rerio*) embryos using different combinations of individual stressors, including α-particles and cadmium (Cd) [12,13,14], and α-particles and depleted uranium (DU) [15,16]. The results obtained using α-particles and Cd demonstrated that the multiple stressor effects depended on the magnitudes as well as sequences of application of the individual stressors [12,13,14], e.g., a priming low α-particle dose antagonized the effect of a challenging high Cd dose [12], while a priming low Cd dose antagonized the effect of a challenging high α-particle dose [13], and a simultaneous exposure to high doses of α-particles and Cd led to additive and synergistic effect, with the additive effect likely a manifestation of the weakly synergistic effect [14]. On the other hand, the results on combined effects of α-particles and DU [15,16] are summarized in Figure 2 to help readers understand the underlying classification of different multiple stressor effects more easily.

Nonlinear J-shaped or inverted U-shaped dose-response curves represent the general biphasic hormetic response (hormesis) exhibiting a low-dose stimulation and a high-dose inhibition [17]. The “responses” from multiple stressors and individual stressors are represented by the *y*-axis in Figure 2. The dose regime corresponding to effects below the spontaneous level is referred to as the hormetic zone. Since the “responses” from individual stressors as well as multiple stressors can now be positive (within “hormetic zone”) or negative (outside “hormetic zone”) with respect to the control level, it will be necessary to consider this “direction” in addition to the “magnitude” (absolute value) of the responses with respect to the control level. Classification of the multiple stressor effect can become complicated in presence of the biphasic hormetic response, but can be simplified if the “responses” from both individual stressors are within the “hormetic zone” (as in Condition 1) or both are outside the “hormetic zone” (as in Condition 2). Under such circumstances, the multiple stressor effect will be synergistic, additive or antagonistic if the magnitude of its response is larger than, equal to or smaller than the sum of magnitude of responses from the two individual stressors. As such, conditions 1 and 2 represent antagonistic and additive multiple stressor effects.

However, classification of the multiple stressor effect is more complicated if the “response” from one individual stressor is within the “hormetic zone” (negative response) and the “response” from the other individual stressor is outside the “hormetic zone” (positive response), as in conditions 3 and 4. Under such circumstances, the multiple stressor effect is antagonistic when its response lies somewhere between the responses from the individual stressors (referring to the *y*-axis in Figure 2), in such a way that the value of the response is “less negative” than the negative value of one stressor, and at the same time is “less positive” than the positive value of the other stressor, which is the case for Condition 3. As regards Condition 4, the value of the response of the multiple stressor effect is “more positive” than the positive value of one stressor (high DU exposure), and at the same time is “less negative” than the negative value of the other stressor (low α-particle dose). In other words, the multiple stressor effect is “synergistic” with reference to the high DU exposure, but it is “antagonistic” with reference to the low α-particle dose. As such, we described the multiple stressor effect as “difficult to define”. 

## 2. Background Information on Radon, Cancer Risk and Dosimetry

### 2.1. Radon and Its Progeny

Radon (^222^Rn) is an inert gas which is a decay product of ^238^U in the earth’s crust through the intermediate radionuclide ^226^Ra, and is thus ubiquitous in our environment (e.g., [18,19]). While most of the radon gas inhaled will be exhaled, the short-lived decay products of radon gas (referred to as radon progeny) including ^218^Po, ^214^Pb, ^214^Bi and ^214^Po, can deposit in the human respiratory tract (HRT) (e.g., [20,21,22,23,24,25,26,27,28,29]).

Research interests on radon started from early 80′s in the last century [30], which were mainly due to the enhanced indoor radon concentrations identified as a result of reduction in indoor ventilation rates, which were in turn attributed to the global energy crisis in that period. While outdoor radon concentrations were typically between 5 and 15 Bq/m^3^, the world average indoor radon concentration was estimated to be about 37 Bq/m^3^, and indoor radon concentrations as high as several kBq/m^3^ were also detected [18,31]. In addition, people spent increasingly more time in indoor environments including dwellings and offices, leading to large indoor occupancy factors, and were thus exposed to radon more than previous generations. These are also the reasons why we will only focus on indoor radon in the present review.

The risk of residential radon exposure has become a matter of international concern [32]. It has now been accepted that α-particles from shorted-lived radon progeny contribute the largest natural radiation dose to mankind [29,33], and the radon progeny could account for more than 50% of the total effective dose received from all natural radioactive sources [34,35]. In fact, radon has been widely accepted as the second leading cause of lung cancers after cigarette smoke [36], although it should also be remarked that the relationship between lung cancer risk and radon concentration is in fact non-trivial (see Section 2.2 below).

### 2.2. Cancer Risk

Ionizing radiations emitted from the decay of these short-lived radon progeny, in particular α-particles, will lead to lung tissue damages in the human body. Although most research on radon risk focus on the α-particle dose, there should also be some risk contributed by the β-particles emitted from radon progeny. For example, large β-particle doses from radionuclides inhaled by dogs were found to increase their cancer risks [37,38]. Generally speaking, ionizing radiations can inflict damages to molecules of deoxyribonucleic acid (DNA) in cells in our body, such as single-strand breaks (SSBs), double-strand breaks (DSBs), and base damages [39,40], and the subsequent loss of genetic integrity can lead to mutation and induce cancer. In particular, α-particles have high linear-energy-transfer (LET) values and can deliver large doses locally to promote carcinogenesis in the bronchial and bronchiolar epithelia (see Section 2.3 below) [29,39,41,42,43].

Extensive research has previously investigated the biological effects of radon progeny on humans (e.g., [29,44]), which has revealed that airborne radon progeny could lead to health issues for humans, particularly lung cancers [32,33,45,46,47]. This also explains why radon-induced lung cancer risk has been found to be particularly high among underground uranium miners [48], who are used to work in underground mines in which the radon concentrations are high. However, extrapolation of the risks derived for miners to the general population was not straightforward, since the exposure groups of people as well as the environmental conditions were very different. For example, the uranium miners were mostly adult males who were often smokers, while the general population also included children, women, and elderly people. Moreover, it was common that the atmospheric environments in mines contained large amounts of dust particles, diesel products and other toxic substances, while those in homes were relatively much cleaner. The aerosol-size distributions, which critically affected the deposition or radon progeny in the HRT, were also different in mines and in homes. The much higher radon concentrations in the mines compared to homes also made it dubious to extrapolate the risks for miners to the general population. Despite all these differences, the dose conversion factor (DCF) expressed in terms of the effective dose (mSv) per unit exposure (WLM) was estimated to be ~ 5 mSv/WLM [49].

Epidemiological studies have shown that exposures to relatively low residential radon concentrations (as low as 100 Bq/m^3^) can already enhance the lung cancer risk [50]. A collaborative analysis of individual data from 13 European case-control studies performed in 2005 showed that residential radon was responsible for ~ 2% of all cancer deaths in Europe [32]. After stratifications for age, sex, region of residence and smoking, the risk of lung cancer was found to increase by 8.4% per 100 Bq/m^3^ increase in the radon concentration [32]. The results were commensurate with a separate combined analysis of seven North American case-control studies, in that a linear correlation between radon exposure and lung cancer risk was also found [51]. In addition, a later case-controlled study in Galicia (Spain), demonstrated a significant lung cancer risk even for exposures to radon concentrations ranging from 37 to 55 Bq/m^3^ when compared to exposures to lower radon concentrations [52].

In relation, for the same absorbed dose the relationship between radon exposure and lung cancer risk can be affected by factors such as the exposure, the age at exposure, the age at risk, gender, smoking habits, the presence of other carcinogens and the occurrence of nonspecific inflammation of airways, etc. [19].

However, it is remarked that the excess lung cancer risk induced by low radon concentrations was neither empirically detected nor theoretically demonstrated [53]. On the contrary, the opposite conclusion was supported by various studies. For example, in 1995, Cohen identified a strong trend showing a decrease in the lung cancer rates with increasing radon exposure, which disagreed with the traditional linear-no threshold theory, with a discrepancy in the slope of about 20 standard deviations [54]. In 2003, Becker reviewed the health effects of radon in Central Europe, and concluded that the available data supported a nonlinear human response to low and medium-level radon exposures [55]. In 2008, Thompson et al. carried out a case-control study of lung cancer risk from residential radon exposure in Worcester County (MA, USA). The authors employed two different models to compute adjusted odds ratios (AORs), and found AORs significantly less than 1.0 between 50 and 75 Bq/m^3^ and between approximately 85 and 123 Bq/m^3^, respectively [56]. These studies or review demonstrated that the lung cancer risk induced by low radon concentrations might not be adequately predicted through linear extrapolation in the traditional linear-no threshold theory. In relation, the biphasic hormetic response (hormesis) exhibiting a low-dose stimulation and a high-dose inhibition, as depicted in Figure 2, could be a possible scenario resulting in opposite lung cancer risks by low and high radon concentrations. The issue that different data sometimes led to opposite conclusions was also noted and discussed [57].

Besides induction of lung cancers in human beings, radon gas can also dissolve in blood and subsequently move through the blood circulatory system within the human body. However, lung-cancer risk remains the principal health effect of radon in human beings.

### 2.3. Radon Dosimetry

The lung dose and the effective dose due to inhalation of short-lived radon progeny can be assessed through computational modeling and micro-dosimetric studies, which are now most commonly performed using the human respiratory tract model (HRTM) of the International Commission on Radiological Protection (ICRP) (published in 1994, and referred to as the ICRP66 model in the following) [21]. In particular, the model was developed to calculate doses for workers as well as individuals of all ethnic groups, to derive limits on intakes, to be applicable to radioactive gases and particles, and to take into account the influence of various respiratory tract diseases, smoking habits and different air impurities. The ICRP66 model was a very comprehensive model which summarized information in: Section 2.3.1 morphometry of the HRT, Section 2.3.2 respiratory physiology, Section 2.3.3 radiation biology, Section 2.3.4 deposition of inhaled substances in different sections of the HRT, Section 2.3.5 clearance of deposited substances from the HRT, and Section 2.3.6 radiation dosimetry [21,29].

#### 2.3.1. Morphometry

ICRP assigned four anatomical regions for the HRT, i.e.:

(i) extrathoracic (ET) region;
(ii) bronchial (BB) region which consisted of trachea and bronchi;
(iii) bronchiolar (bb) region which consisted of bronchioles and terminal bronchioles;
(iv) alveolar interstitial (AI) region which consisted of respiratory bronchioles, alveolar ducts and sacs with their alveoli, and interstitial connective tissue.

The dimensions of the HRT were adjusted according to the standard functional residual capacity (FRC). It is remarked here that more realistic lung morphometry such as bifurcation regions of the HRT has been considered by researchers [20,27,28,58], which leads to more accurate results. Most recently, Hofmann et al. further discovered that the doses due to inhaled radon progeny were higher in upper lobes compared to the average bronchial dose for the whole lung, and doses were higher and lower in the right upper lobe and left lower lobe, respectively [59].

#### 2.3.2. Respiratory Physiology

The relevant physiological parameters included total lung capacity (TLC), FRC, vital capacity (VC), dead space (*V*_d_), tidal volume (*V*_T_), ventilation rates (*V*_E_), breathing rate and breathing frequency (*f*_R_). The ICRP66 report gave reference values for Caucasian workers, as well as Caucasian non-workers, including those for children of 3 months, 1, 5, 10 and 15 years old, and those for adults (both male and female). ICRP also proposed directions for adaptation for other ethnic groups.

#### 2.3.3. Radiation Biology

Based on radiobiological considerations, the ICRP66 report concluded that basal cells (present in BB region only) and secretory cells (present in both BB and bb regions, but absent in the AI region) should be included in dose calculations, while lymph tissue and lymph nodes were deemed insensitive to ionizing radiations.

#### 2.3.4. Deposition of Aerosols in Human Respiratory Tract

The ICRP66 model considered deposition in individual anatomical region of the HRT, which was called regional deposition. Activity median aerodynamic diameters (AMADs) of 1 and 1.5 μm were adopted for indoor or outdoor exposure of the general public, and for workplace exposure, respectively.

#### 2.3.5. Clearance Model

The ICRP66 model suggested that materials deposited in the HRT were cleared: (i) into blood through absorption, (ii) to the gastrointestinal tract, and (iii) to regional lymph nodes via lymphatic tubes.

#### 2.3.6. Weighting the Doses

ICRP assumed: (i) equal sensitivity of basal and secretory cells in the BB region, i.e., the dose *D_BB_* in the BB region was given by *D_BB_* = 0.5(*D_BB,bas_* + *D_BB,sec_*), where *D_BB,bas_* and *D_BB,sec_* were the doses for basal cells and secretory cells, respectively; (ii) equal sensitivities (risks) of the BB, bb and AI regions, so the same weighting factor of 0.333 was assigned to all of these regions, with the remaining 0.001 assigned to lymphatic tissues. It is noted here that the first assumption, i.e., equal sensitivity of basal and secretory cells in the BB region, might not be true since Nikezic and Yu revealed that basal cells were more sensitive than secretory cells [60].

All the models described above were implemented in LUng Dose Evaluation Program (LUDEP) [61]. Sensitivity analysis was performed by Marsh and Birchall in such a way that all parameters were varied in reasonable ranges around their best estimated values, while all other parameters were kept constants [62]. The authors found that the depth of the sensitive cells and the thickness of tissues were the most important target-cell parameters. This analysis showed that the dosimetric model produced a DCF of ~15 mSv/WLM (which was larger than the value of 5 mSv/WLM derived from epidemiological studies). Subsequently, one more report on lung dosimetry was published by ICRP as a practical guide for application of ICRP66 [63].

Numerous reports and articles on radon exposure were further published since the ICRP Publication 66 was published in 1994, which were surveyed in several reports. For example, the BEIR VII Report (2006) stressed that radon played a main role in environmental exposure [64]. A comprehensive survey (more than 100 pages) on radon was given in the Annex E of Volume II of the UNSCEAR 2006 Report [65]. In particular, epidemiological studies on both domestic and workers (miners) were documented in detail in this report. Subsequently, in the UNSCEAR 2008 Report, the annual average dose from radon was estimated as 1.26 mSv, with the typical range of individual doses as 0.2–10 mSv ([66], p.4, Table 1 in the report). It was emphasized that the dose could be much larger in some dwellings, and the dose would depend on many factors such as the radium concentration in the soil underneath the house and in the construction material, the architectural style, and habits of inhabitants etc. The radon issue was also considered in many places in the UNSCEAR 2017 Report [67]. Here, different types of epidemiological studies were discussed (p. 21–23 in the report). Shortcomings of some studies were also noted, e.g., medical doses were not considered in the course of exposure estimation, which could lead to overestimation of radon risks.

### 2.4. Background for Focus in Present Review

In the present review, we will only focus on indoor radon, since indoor radon gas concentrations are generally much higher than outdoor radon concentrations, e.g., reaching a factor of ~5 [18], mainly due to the poor ventilation rate, and the much larger indoor occupancy factor than the outdoor occupancy factor.

Studies on the effects of combined exposures to radon and cigarette smoke in adults have been an interesting and important research direction. Exposures to cigarette smoke can lead to asthma and allergic symptoms. Asthma is associated with chronic lung inflammation, where the airway is reversibly narrowed, which can cause symptoms such as wheezing, cough and shortness of breath [68]. Moreover, chronic obstruction of lung can also affect the bronchial morphometry (see Section 5.1 below). Interestingly, smoking-induced asthma with the chronic obstruction in the lungs could affect the deposition of radon progeny in the lungs to synergistically increase the radon-induced lung cancer risk [69,70].

## 3. Background Information on Phthalates and Effects on Human Respiratory Health

As highlighted in the Introduction, when discussing the effects of airborne contaminants in realistic indoor environments, the effects from collective exposures to all significant airborne contaminants would be relevant, as depicted in Figure 1. Apart from exposures to radon progeny as detailed above in Section 2, people are exposed to various toxic chemicals such as cigarette smoke and phthalates. In fact, concerns have been raised about increased lung cancer risk due to indoor radon, which might have combined health effects with other carcinogenic chemicals and aerosols such as cigarette smoke [71]. While exposures to cigarette smoke as well as phthalates are associated with asthma and allergic symptoms, combined exposures to radon progeny and cigarette smoke are more likely to occur in adult smokers, but not as likely in pediatrics. In fact, as explained below, combined exposures to radon progeny and phthalates are more likely to occur in children. It has been well established that people can be exposed to phthalates through inhalation, ingestion and dermal absorption [72]. Nevertheless, the main objective of the present paper was to provide a review to readers interested in the multiple stressor effects of airborne radon progeny and phthalates, so we would focus on the exposures to phthalates through inhalation. However, it is noted in the outset that previous studies described in the current section which linked phthalate exposure to asthma might not only be due to inhalation, but could also be due to ingestion.

**Figure 3:**
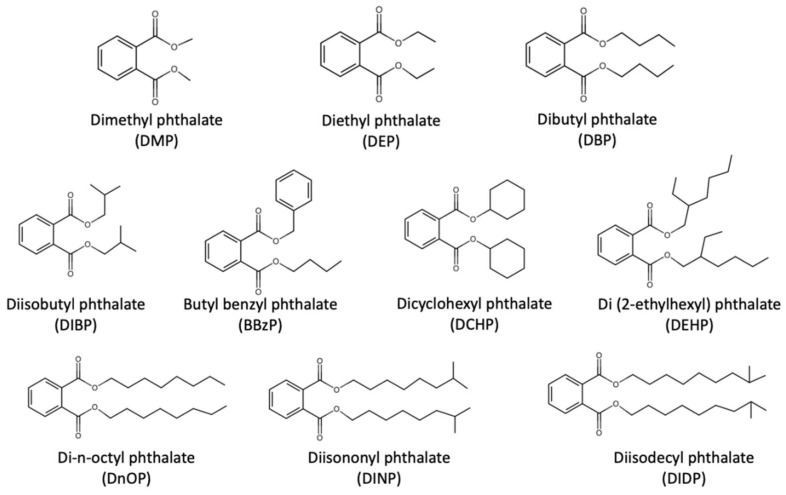
Structures of the ten widely used phthalates summarized by Wang et al. [73].

Phthalates are semi-volatile organic compounds which are widely applied as plasticizers into polyvinyl chloride (PVC) in order to impart flexibility of plastic in consumer products such as toys, flooring materials, wall paper, furniture, building materials, food containers as well as medical devices. Ten widely used phthalates were summarized by Wang et al., including dimethyl phthalate (DMP), diethyl phthalate (DEP), dibutyl phthalate (DBP), diisobutyl phthalate (DIBP), butyl benzyl phthalate (BBzP), dicyclohexyl phthalate (DCHP), di(2-ethylhexyl) phthalate (DEHP), di-*n*-octyl phthalate (DnOP), diisononyl phthalate (DINP), and diisodecyl phthalate (DIDP) [73]. Figure 3 shows the structures of these phthalates. Phthalates are not covalently bound to the polymers so they can easily migrate into the environment with time and use. Strong sources for phthalates have been found in PVC flooring and furniture in home [74]. Furthermore, indoor phthalate concentrations are on average about 10 times more than the outdoor concentrations [75]. In particular, DEHP, BBzP, DBP, DnOP, DEP and DMP have been classified as priority environmental pollutants according to the United States Environmental Protection Agency [76]. In relation, upper limits of 16 ppb and 8 ppb were set for DEHP in surface water [77] and drinking water [78], respectively.

Phthalate metabolites, which are the degradation products of parent phthalates, are commonly used as biomarkers of phthalate exposures [73]. Previous human biomonitoring studies have measured phthalates or their metabolites in human serum, urine, semen, blood and breast milk [79,80,81]. In fact, both parent phthalates and their metabolites could have adverse effects on the human body. Phthalates have been reported to link with endometriosis [82], reduced sperm count and quality [83], decreased testosterone levels [84], metabolic diseases such as diabetes, obesity and breast cancer [85,86,87], as well as allergy and asthma [88]. Braun et al. reviewed the health effect of early life phthalate exposure on pediatrics, in terms of infant size of birth, physical growth, neurodevelopment, genital development as well as childhood asthma and allergy [89]. Regulatory bodies have restricted and banned the use of the phthalates due to their endocrine disrupting effects on human health.

In particular, DEHP which is the most commonly used phthalate accounting for nearly 50% of total global phthalate consumption, is restricted in children’s toy in the European Union [90], the United States [91] and Canada [92]. DEHP was classified as *Possibly carcinogenic to humans* (Group 2B) by International Agency for Research on Cancer working group [93]. As it was established that carcinogenesis was attributed to various endocrine disrupting chemicals (EDCs) such as bisphenol A and dioxin [94], concerns have also been raised on the possible carcinogenicity of phthalates. Although experimental studies demonstrated that phthalate exposure could induce cancer development in murine models [95,96,97], the relationship between phthalate exposure and human carcinogenesis remained unknown due to insufficient and inconclusive human data. It was also noted that no epidemiological studies related phthalate exposure to lung cancer, however, other pulmonary diseases such as asthma was linked with phthalate exposure in existing data [88,98,99,100,101,102,103,104,105,106,107,108,109,110,111,112].

The first epidemiological study on the correlation between interior surface materials and related airway diseases in children was carried out by Jaakkola et al. [98], who revealed that PVC and textile wall materials in homes were linked to development of bronchial obstruction in Norwegian children. In addition to the association between residential PVC products and respiratory symptoms revealed in pediatrics in Norway [98,99], similar associations were demonstrated in Finland [100], Sweden [101,102,103] and Russia [104]. Phthalate-containing dust as well as phthalate metabolites were also linked with the risk of childhood asthma and allergic symptoms [105,106,107,108,109,110,111,112].

In a Bulgarian nested case-controlled study, a significantly higher DEHP level in house dust was found in homes of children who had asthma and allergic symptoms, when compared to homes of children who did not have such symptoms (1.24 vs. 0.86 mg/g dust), and a dose-response relationship was also found between DEHP in dust and allergic symptoms [107]. In a separate study, susceptible periods of development of asthma were identified using longitudinal data. The relationship between PVC flooring in Swedish dwellings for 1- to 5-year-old children with development of asthma was investigated, which was then supplemented with 5- and 10-year follow-up studies [101,102,103]. The 10-year follow-up study showed that children who lived in homes with PVC flooring at 1–5 years of age had larger chance of developing asthma when compared to children who lived in homes without PVC flooring [103]. In another study, the relationships between phthalate exposure and the risk of asthma in children and in adults were reported, where association was shown in adults while no strong evidence was shown in 6- to 17-year-old children [88]. A subsequent study revealed stronger correlations between phthalate concentration in dwellings and risk of asthma in children than in adults in Japan, and proposed that children were more vulnerable to phthalate dust closer to the floor [105]. In relation, there was also a research reporting indoor phthalate exposures to infants and toddlers via inhalation were 12-fold and 6-fold, respectively, larger than those of adults [113].

Phthalate toxicity was also studied through biomarkers and molecular biology. A study in 2012 examined 6- to 9-year-old schoolchildren in New York City and found significantly stronger correlation between urinary phthalate-metabolite levels and fractional exhaled nitric oxide (FeNO; well-established biomarker for airway inflammation) in children with wheeze [114]. In general, humans exposed to environmental concentrations of DEHP have been related to DNA damages [115]. In an in vitro study, induction of inflammatory response in the human lung epithelial cell line A549 by DEHP was confirmed by upregulation of proinflammatory cytokines interleukin-6 (IL-6) and interleukin-8 (IL-8) [116]. A similar effect was observed in A549 cells treated with mono-2-ethylhexyl phthalate (MEHP), which was a metabolite of DEHP [117]. A separate in vitro study showed that inhibition of cell cycle progression, increased apoptotic cell as well as DNA demethylation were induced by DEHP in human bronchial epithelial cells (16HBE cells) [118]. In relation, aberrant DNA methylation was suggested as a mechanism for inactivation of certain tumor suppressor genes in lung cancers [119].

## 4. Lung Cancer Risk of Combined Exposure to Indoor Radon and Phthalates in Children

The present review focused on the potential lung cancer risk of combined exposure to indoor radon and phthalates in children. The focus on indoor contaminants was due to that contaminants in general had higher concentrations in indoor environments (e.g., dwellings, schools and offices) when compared to those in outdoor environments, while at the same time people in general spent most of their time in indoor environments and most of their time were spent in homes (58–69%) [120]. As such, the indoor human health risk could be more significant [121].

On the other hand, the focus on the pediatric population was due to the inherent sensitivity of children, and that children spent large fractions of their time at home, particularly for newborns and preschool children. Children can be subjected to larger exposures under the same environmental conditions due to their physiology and behavior, e.g., their larger body surface-to-volume ratios, unmatured immune systems and hand-to-mouth actions, and as such, they are considered a vulnerable population and prone to higher risks [122]. In addition, as described above, children who spent a much longer time closer to the floor were more vulnerable to phthalate dust [105], and indoor phthalate exposures to infants and toddlers via inhalation were 12-fold and 6-fold, respectively, larger than those of adults [113].

As described in Section 2 and Section 3 above, radon and phthalates are ubiquitous contaminants in our ambient environment. From those discussions, it becomes apparent that people including children will experience exposures to both α-particles (from radon progeny) and phthalates. While the radon-induced lung cancer risk is relatively more studied, the potential risk of phthalate-induced lung cancer is largely unexplored.

## 5. Future Research Directions

### 5.1. Computational Modeling and Micro-Dosimetric Studies

It was suggested that the carcinogenic effects of radon should be taken into consideration together with other inhaled substances rather than radiation alone [20]. As described in Section 2.3, the lung dose and the effective dose due to inhalation of short-lived radon progeny can be assessed using the ICRP66 model published in 1994 [21]. In particular, one important aspect of the model development was to take into account the influence of various respiratory tract diseases, smoking habits and different air impurities. As such, the model would be well suited to study the multiple stressor effects of radon and phthalates.

As regards the sub-model in: Section 2.3.1 morphometry, it is understood that chronic obstruction of lung can affect the bronchial morphometry as described in Section 2 above. The bronchial and bronchiolar airway morphometry will control the equilibrium activities of radon progeny on the surface of airway tubes in the HRT, which will in turn affect the probability of α- particles from the radon progeny to hit basal-cell as well as the secretory-cell nuclei in the BB region as well as the bb region, and finally determine the absorbed lung dose [60]. Furthermore, the absorbed fraction of α-particles emitted from the ^222^Rn chain, the distribution of specific energy, as well as the α-particle lineal energy spectra in the sensitive cells of the HRT, and the DCFs will also change with the thickness of mucus layer HRT, which in fact acts as a source of α-particle emitting radon progeny in the HRT [123,124,125,126].

Consideration of more details of lung morphometry such as bifurcation regions of the HRT can provide even more realistic information [20,27,28,58]. For example, increased local accumulation of radon progeny were shown in bifurcation zones particularly in carinal ridges compared to other sites of tubular bronchial regions, which was a result of the concomitant effect of increased deposition and decreased mucociliary clearance [58].The doses absorbed in the BB and bb regions of the HRT were also found to have a redistribution with the use of the bifurcation model [27,28]. Through employing the bifurcation model, the lung cancer risk was highest at tubular sections of the airway with a sufficiently high radon exposure, while it was significantly higher in the bifurcation zone for a relatively low radon exposure [20], the latter being more relevant to environmentally realistic radon exposures. 

**Figure 4:**
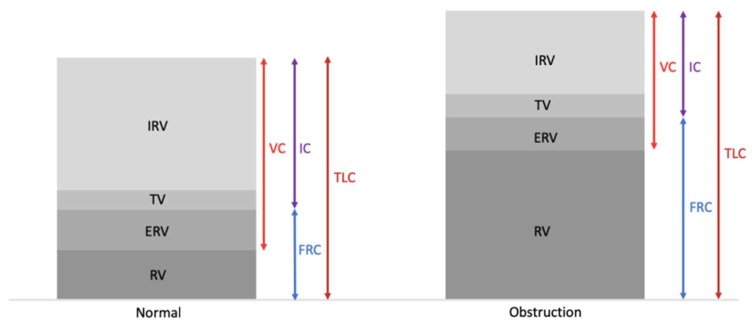
Typical differences in physiological parameters between the normal lung and the lung with obstruction in terms of inspiratory reserve volume (IRV), tidal volume (TV), expiratory reserve volume (ERV) and residual volume (RV) (adopted from [138]).

As regards the sub-model in Section 2.3.2 respiratory physiology, the relevant physiological parameters included TLC, FRC, VC, *V*_d_, *V*_T_, *V*_E_ and *f*_R_. Moreover, reference values were also provided for children of 3 months, 1, 5, 10 and 15 years old. As such, the model would also be well suited to study the multiple stressor effects of radon and phthalates in children. Interestingly, the association between phthalate exposure and lower pulmonary function was confirmed with the measurements of forced vital capacity (FVC), forced expiratory volume in 1 s (FEV_1_) and the peak expiratory flow (PEF) [127,128,129,130,131]. An inverse correlation between phthalate-metabolite levels and PEF was also reported [127,128]. In addition, the increased urinary phthalate-metabolite concentration was shown to reduce FEV_1_ and FVC in adult [127], elderly [129,130] as well as in pediatric population [128,131]. Association was also shown in phthalate-metabolite level and decrease in the FEV_1_/FVC ratio [130,131]. It was noted that a reduced FEV_1_/FVC ratio was used as an indication of airway obstruction in spirometry [132]. It was expected that VC and FVC values were similar for healthy individuals, but would be significantly different for individuals with airway obstruction. It was also confirmed that pulmonary obstruction pattern could cause changes in physiological parameters in the lung, showing positive correlations with FRC, TLC, residual volume (RV) and RV/TLC, and negative correlations with VC, inspiratory capacity (IC) and IC/TLC [132,133,134,135,136,137]. Figure 4 shows typical differences in those parameters between the normal lung and the lung with obstruction [138]. Taken together, the lower pulmonary function induced by phthalate exposure would affect the lung dose and effective dose due to inhalation of short-lived radon progeny.

As regards the sub-model in Section 2.3.4 deposition of inhaled substances, the breathing rate will critically control the regional lung deposition for radon progeny and thus the DCFs [139]. The sensitivity analysis performed by Marsh and Birchall mentioned in Section 2.3 above revealed that the breathing rate was the most important subject-related parameter [62].

As regards the sub-model in Section 2.3.3 radiation biology, the main conclusions were that basal cells and secretory cells should be included in dose calculations. Other phenomena in radiation biology including dose responses (such as effect-specific responses) and non-targeted effects were not discussed in detail. These topics will be discussed in Section 5.2 below. The sub-models in Section 2.3.5 clearance of deposited substances and Section 2.3.6 radiation dosimetry were relatively less relevant for studies on multiple stressor effects of radon and phthalates.

### 5.2. Biological Studies

#### 5.2.1. Effect-Specific Micro-Dosimetric Studies

In many micro-dosimetric studies on the absorbed dose in the HRT due to inhaled radon progeny, the survival or death of the target cells irradiated by α-particles was not taken into account. In reality, some of these target cells would not survive [140,141,142], which should be excluded in the computations of the average absorbed dose. Interestingly, consideration of probabilities for cell-killing by applying the effect-specific track length model [140,141,142], which expressed the probability per unit track length in the cell nucleus (chord length) for cell-killing as a function of the LET of the α-particles, would significantly change this average absorbed dose [143]. As such, it is pertinent to perform more extensive studies on such effect-specific micro-dosimetric models. Development of such models or revision of existing models regarding the absorbed dose in the HRT due to inhaled radon progeny would need more detailed information on radiobiological effects of α-particles, which will be discussed in Section 5.2.2 below, as well as potential influence from non-targeted effects, which will be discussed in Section 5.2.3 below.

To facilitate the investigation on multiple stressor effects of radon and phthalates, the ultimate goals are to establish the dose-response relationship as well to develop effect-specific models for combined exposures to radon and phthalates. The first step will be to study in details the biological effects of phthalates, which will be discussed in Section 5.2.4 below. The final step is to derive a separate effect-specific model for the multiple stressor effects of radon and phthalates, which will be discussed in Section 5.2.5 below.

#### 5.2.2. Radiobiological Effects of α-Particles

It has been well established that ionizing radiations can produce biological effects in living organisms initiated by induction of DNA damages in terms of SSBs, DSBs and base damages [40]. In response to those DNA damages, the corresponding cells may undergo repair or cell cycle arrest, or if the damages are substantial, they may undergo programmed cell death through apoptosis. Failures to perform correct repairs can cause gene mutation or chromosomal changes in the surviving progeny, which may then lead to carcinogenesis [144]. In the present review, we only focus on indoor radon and phthalates, and it has been widely accepted that the main health hazard induced by indoor radon can be attributed to the α-particles emitted by the radon progeny. As such, in this section, we will focus on the biological effects of α-particles on the human lung, including the possible pathways to initiate lung cancer progression.

The effects of α-particles on the regulation of gene expression in lung tissues or cells were reported [145,146]. In particular, in a study involving normal human lung fibroblast HFL-1 cells irradiated with α-particle doses in the range from 0 to 1.5 Gy, 208 genes were observed to be dose-responsive, among which 32% were upregulated while 68% were downregulated [145]. In a separate study involving α-particle-irradiated A549 cells, 590 genes were distinctly expressed, and the genes were shown to be dose-responsive, time-responsive as well as both dose- and time-responsive [146]. The gene expression profile obtained in that research study also suggested that α-particle irradiation might inhibit DNA synthesis and mitosis, which led to cell-cycle arrest [146].

There were also numerous studies on the effects of α-particles or radon on lung cells or lung tissues. For examples, malignant transformation was reported in human bronchial epithelial BEP2D cells irradiated by α-particles [147,148], through studying biological endpoints such as growth kinetics, serum-induced terminal differentiation and tumorigenicity. The biological effect was demonstrated even for a single α-particle dose as low as 30 cGy [148].

Lung oncogenesis was observed in rodents subjected to radon exposure through inhalation [45,46,47]. Collier et al. examined the effects of dose and dose rate from inhaled radon on lung carcinogenesis, and the results indicated that the lung cancer risk was elevated with increasing exposure rate at low cumulative exposures, while the lung cancer risk decreased with increasing exposure rate at higher cumulative exposures (>50 WLM) [45]. Chameaud et al. confirmed lung carcinogenesis in rats exposed to radon and its progeny through inhalation with different cumulative doses [46]. Morlier et al. found elevated lung-cancer incidence in 3-month-old male rats exposed to domestic radon through inhalation, and also revealed the association between lung-cancer incidence and the radon-exposure dose rate [47]. With respect to epigenetics, Huang et al. corroborated that aberrant DNA methylation played a critical role in malignant transformation in human bronchial epithelial BEAS-2B cells exposed to radon [70].

As regards the dose-response relationships of α-particles, it has been a common practice for radiation protection purposes to adopt the linear no-threshold (LNT) model, which has effectively assumed that the risk is linearly proportional to the dose, and that there is no threshold dose for the emergence of the risk. However, accumulating evidence has shown that these assumptions are not true. A good example is the biphasic hormetic response (hormesis) which is characterized by the nonlinear J-shaped or inverted U-shaped dose-response curve, i.e., opposite dose responses at high and low doses [17]. The dose regime corresponding to effects below the spontaneous level was referred to as the hormetic zone. A related phenomenon was the “triphasic” dose-response relationship discovered in 2004 by Hooker et al. in the spleen tissue of pKZ1 mice [149], although the ionizing radiation employed in the study was X-ray photons instead of α-particles. The authors discovered an extra “subhormetic” zone when compared to the biphasic hormetic dose-response curve, which corresponded to ultra-low X-ray doses lower than the doses corresponding to the hormetic zone. Subsequently, Choi et al. also uncovered the triphasic dose-response relationship in 2012 in zebrafish embryos [150], although the ionizing radiation employed in the study was protons instead of α-particles. In a more recent study in 2016, Kong et al. confirmed that the triphasic dose-response relationship could also be induced in zebrafish embryos using X-ray photons, and remarked that the triphasic dose response could be a common phenomenon in living organisms irradiated by X-rays [151]. Interestingly, the authors also discovered that the subhormetic zone could disappear and only a biphasic dose response was displayed when X-ray photons with a different hardness were employed [151].

#### 5.2.3. Potential Influence from Non-Targeted Effects

Non-targeted effects of ionizing radiation refer to phenomena where the radiobiological effects do not occur only in the irradiated cells, and the non-targeted effects of ionizing radiation most relevant to our discussion in the present review include the radiation-induced bystander effect (RIBE) and the associated radiation-induced rescue effect (RIRE), as well as the adaptive response (AR).

**Figure 5:**
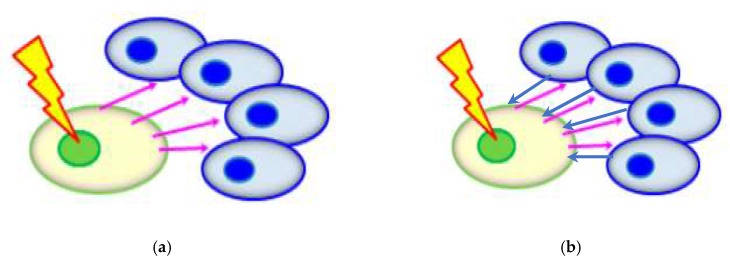
(**a**) Schematic diagram of radiation-induced bystander effect (RIBE). (**b**) Schematic diagram of radiation-induced rescue effect (RIRE). Irradiated cells are shown in green, and bystander cells in blue; bystander signals are shown as pink arrows and rescue signals as blue arrows.

RIBE described the observation that bystander unirradiated cells (non-targeted cells) responded as if they were irradiated upon partnering with irradiated cells (targeted cells) or upon treatment with the medium having previously conditioned the irradiated cells (targeted cells). A schematic diagram of RIBE is shown in Figure 5a. RIBE was first discovered in in vitro experiments [152]. Interested readers are referred to the many reviews on RIBE (e.g., [153,154,155,156,157,158,159]) for further information. As of today, two popular mechanisms have been proposed to explain RIBE, i.e., (1) gap junction intercellular communication (GJIC) when there are physical contacts between the irradiated and bystander cells; and (2) communication of soluble signal factors between the irradiated and bystander cells through the shared medium. The soluble signal factors proposed to participate in RIBE include tumor necrosis factor-α (TNF-α) [160], transforming growth factor-β1 (TGF-β1) [161], IL-6 [162], IL-8 [163] and nitric oxide (NO) [164,165,166] and reactive oxygen species (ROS) [167]. In view of the relevance to our present review, some examples of studies on RIBE involving α-particles are presented here. A significant increase in the sister chromatid exchange (SCE) frequency was observed in bystander HFL-1 cells for an α-particle dose of 0.4 cGy [168]. In the same study, dose dependency of SCE induction was only found in the low-dose range (0.4–2.0 cGy) and not for higher doses (>2.0 cGy). A microbeam study showed that a single targeted cell irradiated by α-particles could lead to on average an additional 100 damaged cells [169]. Elevated expression of the p53 tumor suppressor gene in bystander unirradiated cells were revealed in human diploid fibroblast and rat lung epithelial cells exposed to α-particles [170,171]. Interestingly, it was proposed that the risk from domestic radon exposure would be dominated by RIBE [172].

On the other hand, RIRE described the observation that the harmful effects in irradiated cells (targeted cells) were mitigated upon receiving feedback signals from partnered non-irradiated cells (non-targeted cells), or upon treatment with the medium having previously conditioned the partnered non-irradiated cells (non-targeted cells). A schematic diagram of RIRE is shown in Figure 5b. RIRE was first discovered in in vitro experiments [173]. Interested readers are referred to the recent reviews on RIRE (e.g., [174,175]) for further information. As of today, some mechanisms have been proposed to explain RIRE, including communication of cyclic adenosine monophosphate (cAMP) through a membrane signaling pathway from the bystander cells to the irradiated cells [176] and activation of the nuclear factor-κB (NF-κB) pathway in the irradiated cells [177,178]. These studies involved α-particle-induced RIRE and are thus directly relevant to our present review. Other mechanisms proposed to explain RIRE included the involvement of NO [166,179,180,181,182,183,184,185], induction of autophagy and IL-6 secretion in bystander cells [186] and poly (ADP-ribose) polymerase1 (PARP1) [187]. It is remarked here that there was a different phenomenon (referred to as “Type 2 RIRE” in the review [175]) related to but different from the RIRE first reported in 2011 [173], and was also induced by α-particle irradiation. However, Kong et al. commented that the combination of irradiated/non-irradiated cell types to reveal the “Type 2 RIRE” was different from those used in other studies that displayed traditional RIRE (the one first reported in 2011 [174]) [186].

AR is the phenomenon that a low preceding priming dose of radiation or some chemicals in cells or animals decreases the biological effectiveness of a subsequent high challenging dose [188]. AR in cells was first revealed by Olivieri et al. [189], who reported that peripheral blood lymphocytes pre-irradiated with tritiated thymidine showed fewer chromosomal aberrations when they were irradiated with 15 Gy of X-rays. The successful demonstration of AR had stimulated immense interests in its relationship with radiosensitivity and cancer risk (see e.g., [190]). Upon exposure to a large radiation dose, radioresistant individuals tend to respond in a more beneficial way as a result of enhanced repair processes triggered by AR (see e.g., [191]). Preston reviewed studies on radiation- and chemical-induced ARs as well as the underlying mechanisms, and highlighted the importance of using appropriate mechanistic data in the estimation of the cancer risk from those exposures at low or environmental levels, instead of extrapolating linearly from human tumor data alone [192].

Most traditional studies employed radiations with low LET, e.g., X-ray photons, to deliver the priming dose to induce AR both in vitro and in vivo. In relation, there were reports that high LET radiation could not induce AR in cell cultures [193,194]. Interestingly, our previous studies reported that a priming dose delivered by α-particles which was a high LET radiation could induce AR against a challenging dose also delivered by α-particles in zebrafish (*Danio rerio*) embryos [195,196,197]. These results were particularly relevant to studies on the multiple stressor effect of radon (α- particles) and phthalates in children, and the associated potential cancer risk. Induction of AR was also successfully demonstrated using combinations of different ionizing radiations delivering the priming and challenging doses, e.g., proton priming dose against X-ray challenging dose [198,199,200,201], or even combinations of different stressors delivering the priming and challenging doses, e.g., cadmium priming dose against α-particle challenging dose [13], in zebrafish (*Danio rerio*) embryos. Involvement of combinations of different ionizing radiations or different stressors was directly relevant to studies on the multiple stressor effect of radon and phthalates in children.

#### 5.2.4. Biological Effects of Phthalates

Toxic and genotoxic effects of phthalates were reported. The genotoxicity of DEHP reported by different in vitro and in vivo studies was reviewed by Caldwell, with human and animal data, in terms of induction of DNA lesions, defective regulation of mitotic rate, apoptosis and cell proliferation, increase of proliferation, tumor mobility, and invasiveness of tumor cell lines, and activation of various nuclear receptors, which could contribute to cancer progression [115]. On the other hand, the toxicity and mechanisms underlying carcinogenesis of DEHP in different targeted organs were reviewed by Rusyn and Corton, with a focus on the liver [202]. These authors also suggested that phthalate-induced multiple molecular signals and pathways rather than a single molecular event contributed to carcinogenesis [202].

Besides DEHP or its metabolite MEHP, other phthalates such as BBzP, DBP and DIBP were also found to be genotoxic, which could contribute to cancer progression. For examples, DBP and DIBP were found to inflict DNA damages in two human epithelial cells [203]. BBzP, DBP as well as DEHP exposure were found to suppress apoptosis in human breast cancer cells (MCF-7) [204]. At the same time, DBP and DEHP (10 µM), and BBzP (100 µM) were shown to significantly enhance cell proliferation of MCF-7 cells [204].A separate study also showed that BBzP and DBP (10^−8^–10^−5^ mol/l), and DEHP (10^–8^–10^–6^ mol/L) significantly enhance cell proliferation of MCF-7 cells [205].

There were also numerous reports on the toxic and genotoxic effects of phthalates specifically on the lung cells or tissues, and on the associated carcinogenic effects. A significant increase in the SCE frequency was found in hamster lung fibroblast V79 cells upon exposures to MEHP (25 and 50 µg/mL) for 24 h [206]. In the same paper, a similar study with a shorter exposure time (3 h) revealed that significantly increased SCE frequency was only observed for larger concentrations of MEHP (1500 µM) (no significant effects for concentrations of 750 and 1000 µM) [206]. In a separate study, DEHP exposure was verified to alter cell proliferation, cell cycle progression and apoptosis on 16HBE cells [118]. Ma et al. proposed that inhibition of cell proliferation in DEHP-exposed 16HBE cells might result from disruption of cell cycle progression and accelerated apoptosis. In the same study, DEHP was also found to reduce the degree of global DNA methylation levels, with confirmation of decreased expression levels of DNA methyltransferases (DNMTs), which highlighted potential epigenetic effects of DEHP [118].

On a separate note, invasion and migration of lung cancer cells were successfully demonstrated in A549 and H1299 lung cancer cells with DEHP exposure [116,207] The viability of A549 cells was significantly enhanced upon exposure to DEHP even as low as 5 µM [207]. A similar effect was observed in DEHP-treated H1299 cells. Although no significant effects were found in A549 and H1299 cells with lower DEHP exposures (<10^−4^ M), nanomolar DEHP had stimulated in vitro migration and invasion of both lung cancer cells via upregulation of IL-6 mediated by the NF-κB signaling pathway [116].

There were also a limited number of studies on the toxicity of phthalates in the lungs of rodents [95,96,208,209,210]. In a study involving 9-week-old Wister rats through inhalation exposure to DEHP (aerosols) for 28 days, a statistically significant (16%) increase in the relative lung weight with increased foam-cell proliferation and thickening of the alveolar septi in male rats in the largest-dose group (230 mg/kg/day) was reported [208]. In a similar study involving F344 rats through dietary feeding study to DEHP for 104 weeks, significantly higher mean relative lung weights were reported in male rats treated with 2500 and 12,500 ppm DEHP [95]. David et al. further reported a similar effect in male B6C3F1 mice through dietary DEHP exposure for 104 weeks in the largest-dose group (6000 ppm) [96]. On the other hand, the toxicity of perinatal and postnatal exposure of phthalates were also studied in rats [209,210]. Newborn rats received DEHP injection (750 mg/kg/day), and a significant decrease in the radial alveolar count was found in 14-day-old rats but not in 7-day-old rats, which suggested that prolonged-postnatal exposure of DEHP could lead to inhibition of lung alveolarization and delayed lung development [209]. Perinatal exposure of DEHP to newborn rats caused postnatal growth restriction, and a significantly increased lung interstitial tissue proportion was found for the high-dose group (750 mg/ka/day), which indicated the reduction of gas-exchange space [210].

As regards the dose-response relationships of phthalates, the interesting “triphasic” dose-response relationships discovered for ionizing radiations (X-ray photons and protons) [149,150,151] and described above in Section 5.2.2 were also noted for phthalates. In 2006, Andrade et al. investigated the effects of DEHP exposure on aromatase activity in Waster rats and reported dose-response relationships compatible with triphasic dose-response relationships, although the data in the subhormetic zone were not significantly different from the spontaneous levels [211]. In a most recent study in 2020, Yuen et al. studied the effect of environmentally realistic concentration of DEHP (0–10 ppb) on 9-day old Japanese medaka embryos and also reported dose-response relationships compatible with triphasic dose-response relationships [212]. Specifically, significant mortality was observed in medaka embryos exposed to ultralow dose (0.001 ppb) and high dose (10 ppb) of DEHP, while no significant mortality was noticed in low to medium doses (0.01–1 ppb). The study also attributed chronic effects to short-term low-level environmental DEHP exposures during early development of the Japanese medaka (from 4 h post-fertilization (hpf) to 21 days old) [212]. The authors also speculated that the high sensitivity of medaka embryos to DEHP might be attributed to rapid development of their organs [212], which rendered the results particular relevant when discussing the biological effects of phthalates in children.

#### 5.2.5. Multiple Stressor Effect of Radon (α-Particles) and Phthalates

Establishment of the dose-response relationship and development on effect-specific models for combined exposures to radon and phthalates are indispensable for facilitating investigations on multiple stressor effects of radon and phthalates, as outlined in Section 5.2.1 above.

As described in Section 1 above, Ng et al. examined the multiple stressor effects of α-particles and depleted uranium on zebrafish embryos [15,16], and identified the presence of additive effect, antagonistic effect as well as a “difficult-to-define” multiple stressor effect. The multiple stressor effects were already sophisticated as shown in Figure 2, by taking into account the biphasic hormetic dose-response relationship for both α-particle irradiation and depleted uranium exposure. In relation, the authors also attempted to explain the multiple stressor effects in terms of promotion of early death of cells predisposed to spontaneous transformation by α-particles and at the same time delay in cell death caused by depleted uranium exposure.

Now that triphasic dose-response relationships have been discovered for both ionizing radiations and phthalates, even more sophisticated dose-response relationships could be anticipated, and attempt to explore the underlying explanations could be even more challenging. In the same research [15,16], the authors stressed the importance of examining the multiple stressor effects from individual stressors at both low and high doses in view of the biphasic characteristics of the dose-response relationships (see Figure 2). With the advent of the triphasic dose-response relationships, it becomes apparent that the multiple stressor effects should be determined from individual stressors at ultra-low, low as well as high doses.

## 6. Summary and Discussion

The present paper has reviewed background information available for helping study multiple stressor effects of radon and phthalates in children, and has provided insights on future directions in this research area. In realistic situations, living organisms are subjected to various environmental stressors collectively. The resultant effects are referred to as multiple stressor effects. Section 1 gave a brief introduction to the multiple stressor effect.

Radon is an inert gas which is a decay product of ^238^U in the Earth’s crust through the intermediate radionuclide ^226^Ra, and is thus ubiquitous in our environment. Radon has been widely accepted as the second leading cause of lung cancers after cigarette smoke, although the relationship between lung cancer risk and radon concentration is non-trivial (see Section 2.2). Section 2 reviewed basic properties of radon and its progeny, radon-induced cancer risk and radon dosimetry. As regards radon dosimetry, the comprehensive HRT model (HRTM) for Radiological Protection published by ICRP in 1994 as their Publication 66 was reviewed. Information on: Section 2.3.1 HRT morphometry, Section 2.3.2 respiratory physiology, Section 2.3.3 radiation biology, Section 2.3.4 deposition of inhaled substances in different HRT sections, Section 2.3.5 clearance of deposited substances from the HRT, and Section 2.3.6 radiation dosimetry was summarized.

Phthalates are semi-volatile organic compounds commonly employed as plasticizers to give flexibility of plastic in consumer products, and are also ubiquitous contaminants in our ambient environment. Phthalates have been confirmed to link with endometriosis, reduced sperm count and quality, decreased testosterone levels, metabolic diseases such as diabetes, obesity and breast cancer, as well as allergy and asthma. Section 3 reviews background information on phthalates and their effects on human respiratory health.

Section 4 further provided the rationale behind the current focus on the risk of collective exposure to indoor radon and phthalates in children. The focus on indoor contaminants was due to their higher concentrations and the higher indoor occupancy factor, while the focus on the pediatric population was due to their inherent sensitivity and the fact that they spent more time close to the floor.

Section 5 proposed two major potential future directions in studying multiple stressor effects of radon and phthalates in children. The first one was on computational modeling and micro-dosimetric studies, a major part of which would need the ICRP HRTM. For example, changes in the bronchial and bronchiolar airway morphometry, thickness of mucus layer and the breathing rate due to phthalate-induced asthma can lead to changes in the deposition pattern of radon progeny in the HRT, and will therefore affect radon-induced lung cancer risk. Lower pulmonary function induced by phthalate exposure would also have a similar effect. Taking into account more details of the HRT morphometry such as bifurcation regions can yield even more realistic information. The second proposed major potential future direction is on biological studies. In particular, dose-response relationship as well as effect-specific models for combined exposures to radon and phthalates would be necessary. Development of such dose-response relationship and effect-specific models would need detailed information on biological effects of α-particles and phthalates, potential influence from non-targeted effects such as radiation-induced bystander effect and radiation-induced rescue effect.

Although the focus of the present review was on multiple stressor effects of radon and phthalates in children, the ideas and methodology behind the proposed future research work outlined in Section 5 are also applicable to studies on multiple stressor effects of collective exposures to other significant airborne contaminants, and also to population groups other than children.
